# Advancing Through Innovation: Oral Health Continuing Education for Behavioral Health Providers

**DOI:** 10.1002/jdd.70026

**Published:** 2025-09-03

**Authors:** Adrienne Lapidos, Jennifer Cullen, Elena Flores, Danielle Rulli

**Affiliations:** ^1^ Department of Psychiatry University of Michigan Medical School Ann Arbor Michigan USA; ^2^ Division of Dental Hygiene University of Michigan School of Dentistry Ann Arbor Michigan USA; ^3^ University of Michigan School of Social Work Ann Arbor Michigan USA; ^4^ The Ohio State University College of Dentistry Columbus Ohio USA

## 1 Problem

The intertwined relationship between mental health (MH) and oral health is well established [1]. MH and substance use disorders (SUD) increase the risk of poor oral health; for example, with triple the odds of edentulousness [2, 3]. Avolition, which impacts oral self‐care, and psychiatric medication side‐effects, such as xerostomia, are possible mechanisms driving observed disparities [2, 3, 4]. Yet many behavioral health professionals (BHPs), such as social workers and psychiatrists, have limited knowledge of oral health. The de‐prioritization of oral health in the US health policy may send a message to non‐dental providers that dental care is inessential [5].

## 2 Solution

One strategy to improve the oral health of people with MH and SUD is to improve the oral health literacy of BHPs. Empowered BHPs can ask about oral health, refer and advocate for dental care, and provide basic oral health education.

Supported by a grant from the Delta Dental Foundation, our team conducted a needs assessment, then developed a 2‐h continuing education (CE) course offered through the University of Michigan School of Social Work (UM‐SSW). Working with a Community Advisory Board (CAB), including people with lived experience of MH or SUD, helped to ensure responsiveness to community needs.

## 3 Results

The needs assessment consisted of surveys (*N* = 57), focus groups (*N* = 14), and interviews (*N* = 4) of social workers, psychiatrists, nurses, and other BHPs. Over half of those surveyed reported they had received 0 h of oral health education, and only 3% strongly agreed that they knew “what to say when bringing up oral health.” A staggering 45% reported rarely or never discussing tooth or mouth pain with patients. Qualitative data revealed that while BHPs came to realize the value of oral health when prompted, few had prioritized it.

Responding to these gaps and needs, the CE course included three main themes: (1) “No mental health without oral health:” the burden of oral disease among people with MH and SUD, (2) “Oral health 101:” the oral‐systemic link and key terms like gingivitis and periodontitis, and (3) “A call to action:” the BHP role in oral health promotion, and resources for everyday practice.

UM‐SSW offered the virtual course synchronously in December 2024. Of the 35 learners, 33 participated in the evaluation (Table 1). The majority of respondents identified as female, reflecting a known gender imbalance among BHPs. A total of 73% reported zero prior hours of oral health education. Most items on the pre–post course evaluation showed improvement in oral health knowledge, prioritization, and practice (Table 2). The vast majority reported they intended to change their practice after the course (Table 3).

**TABLE 1:** Learner demographics (*N* = 33).

Age	*N* (%)
18–24	0 (0%)
25–34	13 (40.63%)
35–44	10 (31.25%)
45–54	5 (15.63%)
55–64	3 (9.38%)
65+	1 (3.13%)
Prefer not to say	0 (0%)
Missing	1 1 (3.13%)
**Sex/gender**	***N* (%)**
Male	0 (0%)
Female	30 (93.75%)
Non‐binary	2 (6.25%)
Other	0 (0%)
Prefer not to say	0 (0%)
Missing	1 (3.13%)
**Race/ethnicity** ^a^	***N* (%)**
American Indian/Native American or Alaskan Native	0 (0%)
Asian	0 (0%)
Black or African American	2 (6.25%)
Latino/a or Hispanic	0 (0%)
Middle Eastern or North African	0 (0%)
Native Hawaiian or Other Pacific Islander	0 (0%)
White or Caucasian	26 (81.25%)
Other	1 (3.13%)
Prefer not to say	3 (9.38%)
Missing	1 (3.13%)
**Setting**	***N* (%)**
Suburban	19 (59.38%)
Urban	7 (21.88%)
Rural	4 (12.50%)
Prefer not to say	2 (6.25%)
Missing	1 (3.13%)
**Workplace primary payor**	***N* (%)**
Medicaid/Medicaid health plan	12 (37.50%)
Private insurance	9 (28.13%)
Medicare	3 (9.38%)
Other	5 (15.63%)
Self‐pay	0 (0%)
Prefer not to say	3 (9.38%)
Missing	1 (3.13%)
**Population served** ^a^	***N* (%)**
People with mental health diagnoses	19 (42.22%)
People with substance use disorder diagnoses	6 (13.33%)
People with co‐occurring (MH and SU) diagnoses	14 (31.11%)
Prefer not to say	6 (13.33%)
Missing	1 (3.13%)
**Prior oral health education**	***N* (%)**
0 h	24 (72.73%)
2–5 h	7 (21.21%)
> 10 h	2 (6.06%)

^a^ Multiple selections permitted.

**TABLE 2:** Pre/post‐course evaluation.

	Pre‐course mean (*N* = 33)	Post‐course mean (*N* = 33)	One‐month follow‐up mean (*N* = 18)
How confident do you feel in your knowledge of how oral health and behavioral health are connected?	3.85 ± 2.63 (1–10)	7.56 ± 1.59 (4–10)	7.59 ± 1.24 (6–10)
How confident do you feel that you can help the people you serve, protect, maintain, and improve their oral health?	4.21 ± 2.66 (1–10)	6.74 ± 1.61 (2–10)	6.78 ± 1.58 (3–9)
In your opinion, how high of a priority is oral health to the people you serve, relative to other priorities?	4.61 ± 2.23 (1–8)	5.61 ± 2.47 (1–10)	5.44 ± 2.45 (1–10)
In the past month, how many total conversations about oral health have you had with the people you serve?	3.18 ± 5.81 (0–21^a^)	NA	1.71 ± 2.54 (0–10)
In the past month, how many people have you attempted to help access a dental appointment?	2.12 ± 4.60 (0–21^b^)	NA	0.67 ± 1.15 (0–4)

^a^Two participants endorsed “> 20.” This was imputed as 21 to calculate a mean.

^b^One participant endorsed “> 20.” This was imputed as 21 to calculate a mean.

**TABLE 3:** Intent to change practice.

	Immediate post (*N* = 31)		One‐month follow up (*N* = 18)	
	Yes	No	Yes	No
*Immediate post*: After participating in this course, do you intend to change any aspect(s) of your practice? *One‐month follow up*: So far, did the oral health course have an impact on your practice?	27 (87.1%)	4 (12.9%)	14 (77.78%)	4 (22.2%)

Among the 13% of BHPs who did not intend to change their practice, qualitative responses included “I still don't know how to broach the topic” and “I'm not sure what I would change.” One lesson learned is that a greater emphasis on everyday practice may be necessary to overcoming BHPs' prior lack of oral health education.

Because attending a synchronous class during work hours can be infeasible, UM‐SSW now offers the course on‐demand at https://ssw.umich.edu/r/oralhealth.

Oral health is essential to whole health. This initiative resulted in a widely available CE course for BHPs serving patients who are extraordinarily vulnerable to oral disease, highlighting the potential benefits of interprofessional collaboration in health professions education.

## Conflicts of Interest

The authors declare no conflicts of interest.
